# Acceptability of a Brief Web-Based Theory-Based Intervention to Prevent and Reduce Self-harm: Mixed Methods Evaluation

**DOI:** 10.2196/28349

**Published:** 2021-09-14

**Authors:** Chris Keyworth, Rory O'Connor, Leah Quinlivan, Christopher J Armitage

**Affiliations:** 1 School of Psychology University of Leeds Leeds United Kingdom; 2 Suicidal Behaviour Research Laboratory Institute of Health and Wellbeing University of Glasgow Glasgow United Kingdom; 3 NIHR Greater Manchester Patient Safety Translational Research Centre The University of Manchester Manchester United Kingdom; 4 Manchester Centre for Health Psychology The University of Manchester Manchester United Kingdom; 5 Manchester University NHS Foundation Trust The University of Manchester Manchester United Kingdom

**Keywords:** self-harm, implementation intentions, acceptability, online, volitional help sheet, digital health, mental health

## Abstract

**Background:**

The volitional help sheet (VHS) for self-harm equips people with a means of responding automatically to triggers for self-harm with coping strategies. Although there is some evidence of its efficacy, improving acceptability and making the intervention available in a web-based format may be crucial to increasing effectiveness and reach.

**Objective:**

This study aims to use the Theoretical Framework of Acceptability (TFA) to explore the acceptability of the VHS, examine for whom and under what circumstances this intervention is more or less acceptable, and develop a series of recommendations for how the VHS can be used to support people in reducing repeat self-harm.

**Methods:**

We explored acceptability in two phases. First, our patient and public involvement partners evaluated the original VHS from a lived experience perspective, which was subsequently translated into a web-based format. Second, a representative sample of adults in the United Kingdom who had previously self-harmed were recruited via a YouGov survey (N=514) and were asked to rate the acceptability of the VHS based on the seven constructs of the TFA, namely, *affective attitude, burden, perceived effectiveness, ethicality, intervention coherence, opportunity costs,* and *self-efficacy*. Data were analyzed using descriptive statistics, one-tailed *t* tests, and binary logistic regression. A directed content analysis approach was used to analyze qualitative data.

**Results:**

Participants in the web-based survey rated the VHS as positive (*affective attitude*; *t*_457_=4.72; *P*<.001); were confident using it (self-efficacy; *t*_457_=9.54; *P*<.001); felt they did not have to give up any benefits, profits, or values when using it (*opportunity costs*; *t*_439_=−15.51; *P*<.001); understood it and how it worked (*intervention coherence*; *t*_464_=11.90; *P*<.001); and were confident that it would achieve its purpose (*perceived effectiveness*; *t*_466_=2.04; *P*=.04). The TFA domain *burden* appeared to be an important indicator of acceptability. Lower levels of perceived burden when using the VHS tool were more prevalent among younger adults aged 18-24 years (OR 3.63, 95% CI 1.50-8.78), people of White ethnic background (OR 3.02, 95% CI 1.06-8.613), and people without a long-term health condition (OR 1.53, 95% CI 1.01-2.30). Perceived modifications to further improve acceptability included improved formatting (*burden*), the feature to add new situations and responses or amend existing ones (*ethicality*), and clearer instructions and further detail about the purpose of the VHS (*intervention coherence*).

**Conclusions:**

Our findings show high levels of acceptability among some people who have previously self-harmed, particularly among younger adults, people of White ethnic backgrounds, and people without long-term health conditions. Future research should aim to improve acceptability among older adults, people from minority ethnic groups, and people with long-term health conditions.

## Introduction

### Background

Self-harm is a major public health concern that has a major impact on health care services [1,2] and is growing in prevalence in the United Kingdom [3]. Consequently, the management of self-harm is a widely recognized challenge, and developing preventative strategies is vital [4,5]. Self-harm may include self-harm with suicidal intent (suicidal attempts), self-harm without suicidal intent (nonsuicidal self-harm [NSSH]), or suicidal thoughts [6].

There are many reasons why people engage in self-harm [7] but common among them are triggers, such as feelings of defeat or entrapment [8] that increase the urge to self-harm. Providing people with a means of responding to such critical situations may lessen the likelihood of self-harm. Implementation intentions [9] may be valuable in this regard, as they automatize coping responses to trigger critical situations.

Implementation intentions are *if-then* plans that help people to link a critical situation (ie, *if*) with an appropriate response (ie, *then*). There is a wide body of research illustrating the effectiveness of implementation intentions [10], yet little research has examined the effectiveness of implementing intention-based interventions for reducing repeat self-harm. To help people reduce repeated self-harm, implementation intentions can help people recognize when they feel the urge to self-harm and provide alternative coping strategies. The if-then plans work by making automatic links [10] in memory between a critical situation (“If I am tempted to self-harm when I want to get some attention...”) and an appropriate response (“...then I will do something else instead of self-harming”). Implementation intentions have been shown to be effective in reducing self-harm in people recently admitted to the hospital for self-harm [11]. In this study, participants were provided with a tool, the volitional help sheet (VHS), designed to assist with the formation of implementation intentions to reduce self-harm. The tool provides people with a list of critical situations where the urge to self-harm may be heightened, and a list of responses designed to increase the likelihood of not self-harming [12]. The development of the VHS for self-harm has been described elsewhere [11]. Briefly, the VHS provides a theoretically driven framework for participants to construct their own implementation intentions, drawing on theories of suicidal behavior [13], self-harm motivation literature [14], and the transtheoretical model of change [15]. However, previous research is limited by high rates of attrition at follow-up [11]. Other studies report that the effectiveness of implementation intentions for reducing repeat self-harm may vary as a function of self-harm history [16]. Given the likelihood of successful implementation and effectiveness of interventions may be dependent upon perceptions of acceptability [17,18], it is necessary to comprehensively examine the acceptability of the VHS for self-harm in further detail.

Intervention acceptability is an important consideration in the design, implementation, and evaluation of health care interventions [19,20]. The likelihood of successful implementation and effectiveness may depend on perceptions of acceptability [17,20]. For example, interventions perceived as acceptable by those delivering or receiving them are more likely to result in favorable outcomes, including adherence to treatment programs [18], support for public health policy [17], or acceptance of behavior change interventions [21]. The Theoretical Framework of Acceptability (TFA) [20] is an established guide for assessing the acceptability of interventions. It defines acceptability as “a multifaceted construct that reflects the extent to which people delivering or receiving a health care intervention consider it to be appropriate, based on anticipated or experienced cognitive and emotional responses to the intervention” [20]. The TFA comprises seven domains: (1) affective attitude (how individuals feel about taking part in an intervention), (2) burden (the amount of effort required to engage with an intervention), (3) perceived effectiveness (whether individuals perceive an intervention as likely to achieve its purpose), (4) ethicality (the extent to which an intervention fits with individuals’ personal values), (5) intervention coherence (whether individuals understand an intervention and how it works), (6) opportunity costs (what is given up, such as time, to take part in an intervention), and (7) self-efficacy (how confident individuals are performing the intervention). The advantage of using the TFA, as opposed to more general approaches to investigating acceptability, is that the TFA allows a more systematic assessment of intervention acceptability, and this approach allows researchers to target specific TFA domains in future iterations of interventions (eg, addressing perceived burden of interventions) [22].

### Objective

This study has three specific objectives: first, to evaluate the acceptability of the VHS from a lived experience perspective (patient and public involvement [PPI]) and redevelop the tool according to feedback; second, to examine for whom and under what circumstances the VHS is more or less acceptable; and finally, to develop a series of practice recommendations for how the VHS can be used to support people in reducing repeat self-harm.

## Methods

### Overview

Ethical approval was obtained from the University Research Ethics Committee (ref: 2020-8446-15312), and informed consent was obtained from all participants. The VHS provides people with a list of critical situations where the urge to self-harm may be heightened, and a list of coping responses designed to decrease the likelihood of self-harming [12]. The development of the VHS for self-harm has been described elsewhere [11]. Briefly, the VHS provides a theoretically driven framework for participants to construct their own implementation intentions, drawing on theories of suicidal behavior [13], self-harm motivation literature [14], and the transtheoretical model of change [15].

**Figure 1:**
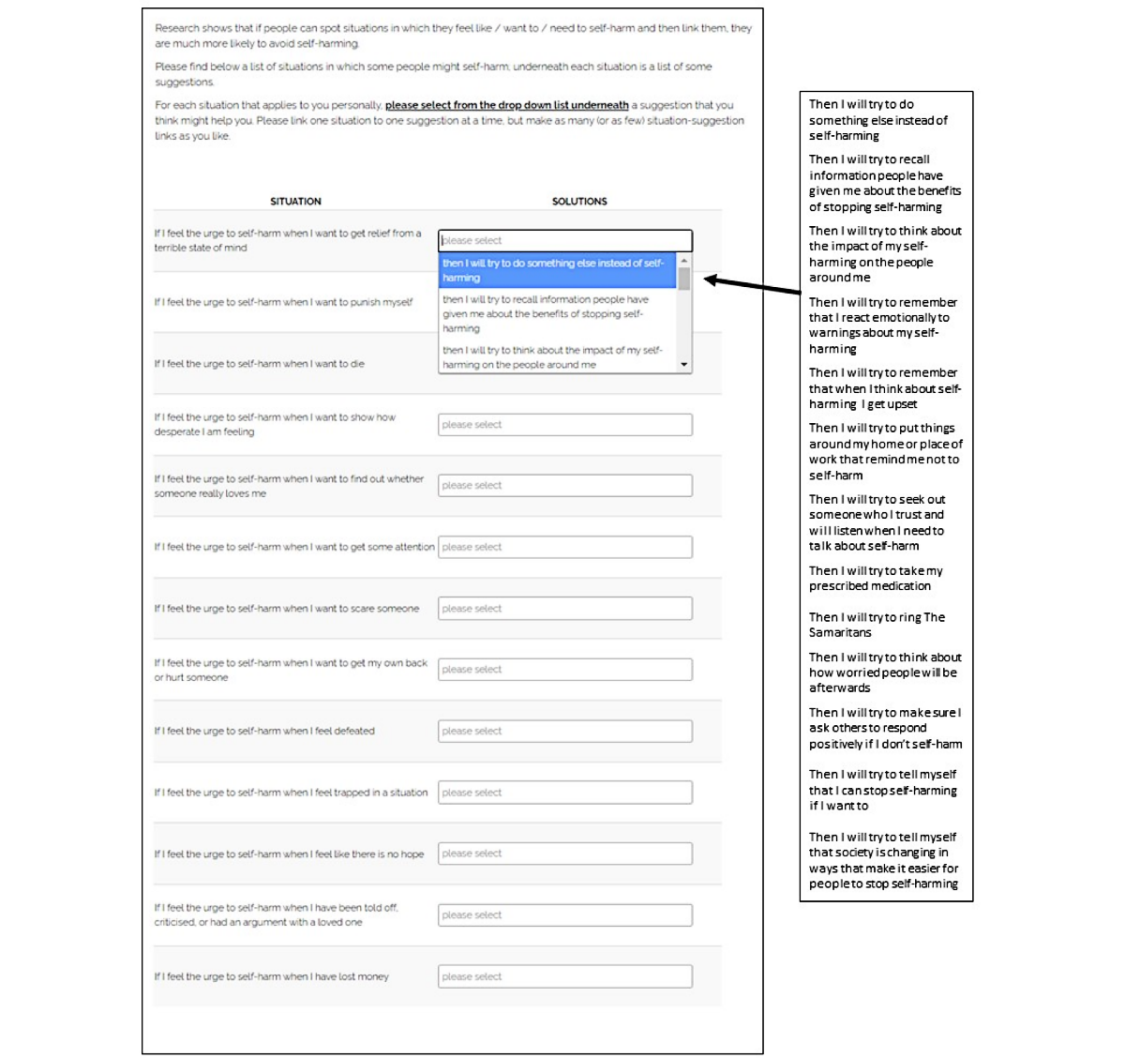
The volitional help sheet for self-harm.

Acceptability was explored in two phases. In phase 1, the original VHS, used as part of a previous study [11], was distributed among a PPI group for initial feedback and translated to a web-based format by the research team. This was done by creating on a single webpage, as part of a web-based questionnaire, a list of situations alongside which participants could choose an appropriate response from a drop-down menu for each critical situation (a screenshot of the VHS is provided for illustrative purposes in Figure 1). As the intervention was presented on a single screen within the questionnaire, participants were able to print the VHS after participation. Participants were also given a physical copy of the VHS as part of the study. In phase 2, a national sample of people in the United Kingdom who had previously self-harmed was recruited via a survey panel company (YouGov), as part of a larger 6-month follow-up study examining the effectiveness of the VHS for reducing self-harm (ClinicalTrials.gov identifier: NCT04420546).

### Phase 1: PPI and Tool Development

The VHS has been shown to be effective for reducing self-harm in people recently admitted to the hospital following an episode of self-harm [11]. The researchers initially made minor changes to the VHS for translation into a web-based format, in line with a previous VHS delivered on the web [23]. To ensure that the intervention was suitable for as broad a population as possible, the VHS was evaluated from a lived experience perspective by members from a PPI group (n=10). The group was specifically trained to provide feedback on research methods and materials to be used as part of intervention delivery, and all members of the group had a history of self-harm, suicidal behavior, or receiving mental health services. The feedback from our PPI contributors was used to ensure that the VHS was understandable and sensitive to people’s thoughts and emotions about self-harm (the majority of the PPI group had a history of self-harm). Participants provided (on hard copies of the VHS) feedback and suggestions for improvements both on the wording of the instructions of the VHS, and the *critical situations* and *appropriate responses.* Previous deployments of the VHS have predominantly used paper and pencil, whereby participants are asked to physically draw a line linking any situations that applied to them, to one solution at a time (participants could make as many situation-solution links as they would like). On the basis of the feedback, amendments were made to the wording of 11 of the situations and nine of the solutions. Two solutions were removed because the group felt the wording was inappropriate (“Then I will tell myself that I can stop self-harming if I want to” and “Then I will make sure I am rewarded by others if I don’t self-harm”). Two new situations (“If I feel the urge to self-harm when I have been told off, criticised, or had an argument with a loved one” and “If I feel the urge to self-harm when I have lost money”), and four new solutions (“Then I will try to ring The Samaritans,” “Then I will try to think about how worried people will be afterwards,” “Then I will try to make sure I ask others to respond positively if I don’t self-harm,” and “Then I will try to tell myself that I can stop self-harming if I want to”) were added to the VHS. The final VHS, which included the two new situations and four new solutions, contained 13 situations and 13 solutions.

### Phase 2: YouGov Survey

A national sample of adults in the United Kingdom who had previously self-harmed were recruited via a survey panel company (YouGov), as part of a larger study (ClinicalTrials.gov Identifier: NCT04420546) were invited to take part in a web-based questionnaire and were incentivized in accordance with the YouGov’s point system (respondents accumulated points for taking part in surveys, which can be exchanged for cash or entry into a prize draw). To ensure that the final sample contained people with a prior history of self-harm, we asked a screen question: “Have you ever intentionally hurt yourself/self-harmed?” Response options were “yes, I have,” “no, I have not,” or “prefer not to say.” The final sample was based on respondents answering, “yes, I have.”

Following completion of the questionnaire (described below), participants were asked to complete the amended VHS to reduce self-harm (based on the feedback obtained from phase 1). Participants formed implementation intentions by linking critical situations with appropriate responses by choosing an appropriate response from a drop-down menu for each critical situation. Participants were free to make as many situation-response links as desired. Participants were then asked questions about their acceptability (n=514).

### Measures

#### Sociodemographic Variables

Demographic variables, including age, gender, ethnicity, and social grade, were taken using the standard UK Office for National Statistics [24] measures.

#### History of NSSH, Suicidal Ideation, and Suicide Attempts

Three items drawn from the British Psychiatric Morbidity Survey [25]: “Have you ever seriously thought of taking your life, but not actually attempted to do so?” (suicidal ideation); “Have you ever made an attempt to take your life, by taking an overdose of tablets or in some other way?” (suicidal attempt); and “Have you ever deliberately harmed yourself in any way, but not with the intention of killing yourself? (ie, self-harm)” (NSSH). Response options for all questions were *Yes*, *No*, or *prefer not to say*. If respondents answered yes to any of the three questions, the timing of the last episode and frequency was asked.

#### Exposure to Death and Suicide

Participants were asked seven items [26,27] to establish whether any of their close friends or family had died, whether they had friends of family who had self-harmed, or who attempted or died by suicide (eg, “Has anyone among your family attempted suicide?”).

#### Acceptability Measures

Only one study has deployed the TFA as a quantitative measure; Renko et al [28] assessed the acceptability of a training program to enhance teachers’ physical activity promotion. However, the items were not sufficiently generic to adapt to the present purposes. Likert scale responses, with additional open-ended text questions to capture qualitative data with respect to each domain, were developed in line with the seven TFA constructs used to assess acceptability. Seven items were developed, for example, “On a scale of 0-10, how much effort was required to use the volitional help sheet?” (burden; *no effort*: 1 to *lots of effort*: 10). Item wordings were developed to closely resemble the definitions provided for each domain of the TFA [20]. The items used to measure each TFA domain are shown in Table 1.

For each of the seven TFA items, participants were invited to provide open-ended comments describing their experiences using the VHS with respect to each of the seven constructs of the TFA. Seven items were developed, corresponding to each of the TFA domains, for example, “Do you have any specific comments about how good or bad you felt when using the volitional help sheet?” (affective attitude). Participants’ comments were combined, and content analysis was performed.

**Table 1:** The Theoretical Framework of Acceptability domains.

Domain	Question	Participant (n=514), n (%)	Values, mean (SD)	Comments (n=360), n (%)
Affective attitude	On a scale of 0-10, how good or bad did you feel when using the volitional help sheet?	476 (92.6)	5.50 (2.32)	33 (9.2)
Burden	On a scale of 0-10, how much effort was required to use the volitional help sheet?	471 (91.6)	5.09 (2.84)	78 (21.7)
Ethicality	On a scale of 0-10, how much was using the volitional help sheet a good fit with your personal values?	465 (90.5)	4.80 (2.68)	69 (19.2)
Self-efficacy	On a scale of 0-10, how confident were you about using the volitional help sheet?	468 (91.1)	6.16 (2.63)	37 (10.3)
Opportunity	On a scale of 0-10, to what extent did you give up any benefits, profits, or values when using the volitional help sheet?	440 (85.6)	2.95 (2.77)	38 (10.6)
Intervention coherence	On a scale of 0-10, how confident were you that you understood the volitional help sheet and how it works?	465 (90.5)	6.45 (2.63)	40 (11.1)
Perceived effectiveness	On a scale of 0-10, how confident were you that the volitional help sheet is likely to achieve its purpose?	467 (90.9)	5.24 (2.59)	65 (18.1)

#### Analyses

Quantitative data were analyzed using SPSS, version 26 (IBM Corporation). Descriptive statistics were used to summarize the sociodemographic variables. The chi-square test was used to compare our sample of people who reported a previous history of self-harm with general population data collected as part of the Adult Psychiatric Morbidity Survey [29]. In the absence of universally agreed criteria for determining acceptability with respect to deploying TFA quantitative measures, two approaches used in previous studies were adopted [30]. First, one-tailed *t* tests were used to assess how far above or below the neutral rating (5) people rated the seven TFA indicators. This measure was used to ensure that the neutral responses were accounted for. Second, the proportion of participants scoring at each point on the rating scale was assessed. These measures were used to assess the desirability and acceptability of the questionnaire according to each of the seven TFA domains. Binary logistic regression was used to explore the correlates of acceptability to identify which sociodemographic factors (gender, age, ethnicity, social grade, and recency of self-harm [past week or past year]; all dummy coded) were associated with higher or lower levels of perceived acceptability. Each of the main outcomes was recorded as a binary outcome (eg, high acceptability: 1 or low acceptability: 0). High acceptability was defined as scores above the neutral rating (ie, scores >5), and low acceptability was defined as scores ≤5, except for two domains (*burden* and *ethicality*) where high acceptability was defined as scores <5, and low acceptability was defined as scores ≥5.

#### Qualitative Analyses of Open‐Ended Comments

Participants were asked to rate the acceptability of the VHS based on the seven constructs of the TFA (described above): affective attitude, burden, perceived effectiveness, ethicality, intervention coherence, opportunity costs, and self-efficacy. Participants were invited to provide open-ended comments to each TFA question. A directed content analysis approach, which is suitable when the research uses an existing theory or framework to interpret the data, was used to identify and categorize instances of the TFA domains [31,32]. First, deductive coding was used to generate a content analysis framework in line with the TFA domains. Second, inductive coding was used to generate explanatory themes with respect to each TFA domain, whereby specific codes within each TFA domain were grouped into themes. Initial codes were generated and collated into potential themes by CK, who shared the coding framework and key illustrative quotes with CJA as the analysis progressed. Any areas of contention were discussed, and themes were refined accordingly to ensure the trustworthiness of the data. All the authors were involved in finalizing the main themes. NVivo, version 12 (QSR International) was used to organize the data. The codes focused on different aspects of acceptability with respect to using the VHS to reduce repeat self-harm, according to each TFA domain (eg, attitudes toward the intervention, and the perceived effort required to engage with the intervention). The themes were reviewed by coauthors, and there were no disagreements.

## Results

### Sample Characteristics

#### Overview

The total sample (n=514) comprised mostly women (331/514, 64.4%), 27.4% (141/514) were aged 18-34 years, 21.2% (109/514) were aged 35-44 years, 18.1% (93/514) were aged 45-54 years, and 33.3% (171/514) were aged ≥55 years. The majority of the sample was White (472/514, 91.8%), and 63.4% (326/514) were of higher social grade (nonmanual workers; Table 2). The characteristics of our sample closely resembled the characteristics of people who reported a history of self-harm according to the Adult Psychiatric Morbidity Survey of the general population [29] in terms of gender and age. However, our sample contained a lower proportion of people from a minority ethnic background compared with the national data.

**Table 2:** Sample demographics (N=514).

Variable		Population, n (%)	Value, mean (SD; range)		General population data (%)^a^		Chi-square difference between sample and population		*P* value	
**Gender**		N/A	N/A^b^		N/A		N/A		N/A	
	Women	331 (64.4)			54.5		1.68		.19	
	Men	176 (34.2)			45.5		3.00		.08	
	Other or prefer not to say	7 (1.4)			N/A		N/A		N/A	
**Age (years)**		N/A	45.80 (14.21; 18-77)		N/A		N/A		N/A	
	18-34	141 (27.4)			26.4		0.03		.87	
	35-44	109 (21.2)			17.8		0.28		.59	
	45-54	93 (18.1)			21.1		0.74		.39	
	≥55	171 (33.3)			34.6		0.09		.77	
**Ethnicity**		N/A	N/A		N/A		N/A		N/A	
	White	472 (91.8)			87.1		1.33		.25	
	BAME^c^	16 (3.1)			12.9		6.79		.01	
	Prefer not to say	26 (5.1)			N/A		N/A		N/A	
**Social grade**		N/A	N/A		N/A		N/A		N/A	
	Nonmanual worker	326 (63.4)			N/A		N/A		N/A	
	Manual or unemployed	188 (36.6)			N/A		N/A		N/A	
**Suicidal ideation (ever)**		390 (75.9)	N/A		20.6		60.55		<.001	
	Past week	38 (7.4)			N/A		N/A		N/A	
	Past year	126 (24.5)			5.4		15.69		<.001	
	Longer ago	221 (43)			N/A		N/A		N/A	
	Would rather not say or did not answer	129 (25.1)			N/A		N/A		N/A	
**Suicidal attempt (ever)**		212 (41.2)	N/A		6.7		31.69		<.001	
	Past week	N/A			N/A		N/A		N/A	
	Past year	N/A			0.7		1.85		.17	
	Longer ago	187 (36.4)			N/A		N/A		N/A	
	Would rather not say or did not answer	303 (59)			N/A		N/A		N/A	
**NSSH^d^ (ever)**		383 (74.5)	N/A		7.3		95.58		<.001	
	Past week	26 (5.1)			N/A		N/A		N/A	
	Past year	68 (13.2)			N/A		N/A		N/A	
	Longer ago	284 (55.3)			N/A		N/A		N/A	
	Would rather not say or did not answer	136 (26.5)			N/A		N/A		N/A	
**Exposure to suicide and death**		N/A	N/A		N/A		N/A		N/A	
	Exposure to death (immediate family)	273 (53.1)			N/A		N/A		N/A	
	Exposure to death (close friend or relative)	390 (75.9)			N/A		N/A		N/A	
	Exposure to death by suicide (family or close friend)	159 (30.9)			N/A		N/A		N/A	
	Suicidal attempt (in the family)	175 (34)			N/A		N/A		N/A	
	Suicidal attempt (by close friends)	189 (36.8)			N/A		N/A		N/A	
	NSSH (in the family)	153 (29.8)			N/A		N/A		N/A	
	NSSH (by close friends)	214 (41.6)			N/A		N/A		N/A	

^a^According to the Adult Psychiatric Morbidity Survey of the general population [29].

^b^N/A: not applicable.

^c^BAME: Black, Asian, and minority ethnic.

^d^NSSH: nonsuicidal self-harm.

#### Prevalence of Suicidal Ideation, Suicide Attempts, NSSH, and Exposure to Suicide and Death

Overall, 75.9% (390/514), 41.2% (212/514), and 74.5% (383/514) of the total sample reported suicidal ideation, suicide attempts, and NSSH, respectively (Table 1). Furthermore, 7.4% (38/514) of the total sample reported suicidal thoughts in the past week, and 24.5% (126/514) of the sample reported suicidal thoughts in the past year. Few people reported suicide attempts in the past week (4/514, 0.8%), and 3.9% (20/514) reported a suicide attempt in the past year. With respect to NSSH, 5.1% (26/514) reported NSSH in the past week, and 13.2% (68/514) reported NSSH in the past year.

Over half of the sample (273/514, 53.1%) reported experiencing the death of a family member, over three-fourths (390/514, 75.9%) of the sample reported experience of the death of a close friend or relative, and 30.9% (159/514) of the sample reported experience of death by suicide of a close friend or relative. Of the total sample, 34% (175/514) reported exposure to a family member making a suicide attempt, and 36.8% (189/514) reported exposure to a suicide attempt by a close friend. Exposure to NSSH by a family member was reported by 29.8% (153/514) of the sample, and NSSH by a close friend by 41.6% (214/514) of the sample.

With regard to lifetime history of self-harm, our sample reported a higher prevalence of suicidal ideation (390/514, 75.9% vs 20.6%), suicide attempts (212/514, 41.2% vs 6.7%), and NSSH (383/514, 74.5% vs 7.3%) compared with national data. With regard to self-harm in the previous year, our sample reported a higher prevalence of suicidal ideation (126/514, 24.5% vs 5.4%) compared with national data; the prevalence of suicide attempts in the previous year more closely resembled national data (0.7% vs 20/514, 3.9%).

#### Overall Acceptability of the VHS

One-sample *t* tests showed that participants rated the VHS favorably on five of the seven indicators by scoring above or below the respective midpoints. Participants rated the VHS positively (*affective attitude*; mean 5.50, SD 2.32; t_457_=4.72; *P*<.001); were confident using the VHS (*self-efficacy*; mean 6.16, SD 2.63; t_457_=9.54; *P*<.001); did not have to give up any benefits, profits, or values when using the VHS (*opportunity costs*; mean 2.95, SD 2.77; t_439_=−15.51; *P*<.001); understood the VHS and how it worked (*intervention coherence*; mean 6.45, SD 2.63; t_464_=11.90; *P*<.001); and were confident that it would achieve its purpose (*perceived effectiveness*; mean 5.24, SD 2.59; t_466_=2.04; *P*=.04). No significant differences were found for the two TFA domains: *burden* (mean 5.09, SD 2.84) and *ethicality* (mean 4.80, SD 2.68). The mean ratings for each of the seven constructs are listed in Table 2.

The proportion of participants scoring at each point on the rating scale of each TFA item is presented in Multimedia Appendix 1. There were two key findings. First, there was a high proportion of responses at the upper end of the *self-efficacy* and *intervention coherence* items, compared with other items (97/468, 20.7% and 110/465, 23.6% of participants, respectively, rating 9 or 10 on the 0-10 scales). Second, there was a high proportion of responses at the lower end of the opportunity costs item compared with other items (186/440, 42.3% of participants rated 0 or 1 on the 0-10 scale).

#### Associations Between Sociodemographic Variables (Age, Gender, Ethnicity, and Current Health Status) and Acceptability

Table 3 shows the binary logistic regression results of perceived acceptability of the VHS according to sociodemographic variables.

No significant differences were found between men and women in perceived acceptability of any of the TFA variables. Lower levels of perceived burden were more prevalent among people aged 18-24 years, compared with those aged 25-34 years (OR 3.63, 95% CI 1.50-8.78), and 35-44 years (OR 2.55, 95% CI 1.06-6.15), and among people of White ethnic background, compared with people from minority ethnic groups (OR 3.02, 95% CI 1.06-8.61). Higher levels of perceived burden were more prevalent among people who reported having a long-term health condition (OR 1.53, 95% CI 1.01-2.30), compared with those who did not report having a long-term health condition.

**Table 3:** Associations between sociodemographic variables and acceptability of the volitional help sheet, according to Theoretical Framework of Acceptability variables.

Variables			*β* (95% CI)												
			Attitude		Burden		Ethicality		Self-efficacy		Opportunity costs		Intervention coherence		Perceived effectiveness
Gender (women)			1.10 (0.75-1.61)		1.17 (0.80-1.71)		1.26 (0.84-1.87)		1.28 (0.87-1.88)		0.91 (0.53-1.58)		1.17 (0.79-1.74)		1.26 (0.86-1.86)
**Age (years; reference group: 18-24)**															
	25-34	0.81 (0.35-1.87)		3.63^a^ *(1.50*-*8.78)*		0.83 (0.36-1.93)		1.51 (0.66-3.45)		0.64 (0.21-2.02)		1.55 (0.67-3.60)		0.82 (0.36-1.87)	
	35-44	0.76 (0.33-1.78)		2.56^b^ *(1.06*-*6.15)*		0.80 (0.34-1.85)		1.72 (0.75-3.96)		0.64 (0.20-2.00)		1.70 (0.73-3.96)		0.78 (0.34-1.76)	
	45-54	1.11 (0.48-2.59)		1.70 (0.69-4.14)		0.73 (0.31-1.73)		1.58 (0.68-3.66)		1.04 (0.34-3.15)		1.31 (0.56-3.07)		0.71 (0.31-1.64)	
	55 or over	1.15 (0.51-2.57)		1.47 (0.63-3.43)		0.86 (0.38-1.94)		1.13 (0.51-2.50)		0.73 (0.25-2.16)		1.17 (0.53-2.61)		0.70 (0.32-1.53)	
Ethnicity (minority ethnic groups)			1.86 (0.72-4.80)		3.02^b^ *(1.06-8.61)*		1.56 (0.59-4.14)		0.77 (0.30-1.98)		2.88 (0.96-8.60)		3.05 (0.87-10.70)		1.51 (0.55-4.16)
Social grade (nonmanual)			0.93 (0.63-1.35)		1.01 (0.70-1.47)		0.92 (0.62-1.35)		1.42 (0.97-2.08)		1.05 (0.61-1.82)		1.21 (0.82-1.78)		1.08 (0.74-1.58)
Long-term chronic health condition			1.16 (0.77-1.74)		1.53^b^ *(1.01-2.30)*		1.29 (0.84-1.98)		1.02 (0.68-1.53)		0.86 (0.49-1.53)		1.10 (0.73-1.67)		0.93 (0.62-1.39)
Marginalized group (yes)			0.75 (0.50-1.11)		1.20 (0.82-1.77)		0.93 (0.62-1.39)		0.90 (0.61-1.33)		1.26 (0.73-2.18)		1.36 (0.90-2.04)		1.16 (0.79-1.72)
Self-harm (past week; any measure)			0.66 (0.37-1.20)		1.55 (0.88-2.72)		1.34 (0.76-2.36)		1.22 (0.69-2.16)		0.72 (0.53-2.48)		0.91 (0.52-1.62)		0.84 (0.54-1.65)
Self-harm (past year; any measure)			0.71 (0.48-1.06)		1.64^b^ *(1.11-2.41)*		0.82 (0.55-1.23)		0.73 (0.50-1.07)		0.78 (0.44-1.38)		1.08 (0.72-1.60)		0.86 (0.58-1.26)
Exposure to death (immediate family)			1.23 (0.85-1.77)		0.62^a^ *(0.43-0.89)*		1.06 (0.73-1.55)		1.02 (0.71-1.48)		1.64 (0.95-2.84)		1.01 (0.70-1.48)		0.93 (0.64-1.33)
Exposure to death (close friend or relative)			1.15 (0.75-1.77)		0.86 (0.57-1.32)		1.11 (0.71-1.73)		0.91 (0.59-1.40)		0.94 (0.51-1.74)		1.25 (0.81-1.93)		0.86 (0.56-1.32)
Exposure to death by suicide (family or close friend)			0.80 (0.54-1.19)		0.97 (0.66-1.44)		1.07 (0.71-1.61)		0.73 (0.49-1.08)		1.06 (0.60-1.88)		0.97 (0.64-1.45)		1.11 (0.74-1.65)
Suicidal attempt (in the family)			*1.53*^b^*(1.04*-*2.25)*		0.95 (0.65-1.39)		1.02 (0.68-1.51)		1.11 (0.75-1.63)		1.32 (0.76-2.27)		1.23 (0.82-1.83)		1.11 (0.75-1.63)
Suicidal attempt (by close friends)			0.87 (0.60-1.28)		1.11 (0.76-1.62)		0.91 (0.61-1.35)		1.20 (0.81-1.76)		1.77^b^ *(1.03-3.03)*		1.73 (0.95-2.14)		1.00 (0.68-1.47)
NSSH^c^ (in the family)			1.13 (0.76-1.69)		1.00 (0.67-1.49)		1.53^b^ *(1.02-2.30)*		1.51^b^ *(1.00-2.27)*		1.04 (0.58-1.85)		2.10^a^ *(1.35-3.27)*		1.33 (0.89-1.99)
NSSH (by close friends)			0.78 (0.53-1.14)		1.33 (0.92-1.94)		1.10 (0.74-1.61)		1.33 (0.91-1.95)		1.30 (0.75-2.24)		1.53^b^ *(1.03-2.27)*		1.02 (0.70-1.48)

^a^*P*<.05.

^b^*P*<.01.

^c^NSSH: nonsuicidal self-harm.

#### Associations Among Prevalence of Suicidal Ideation, Suicide Attempts, NSSH, Exposure to Suicide and Death, and Acceptability

People who reported self-harm in the past year were more likely to report higher levels of burden (OR 1.64, 95% CI 1.11-2.41) than those who did not report self-harm in the past year. No significant differences were found for any of the TFA variables among people who had or had not self-harmed in the past week.

People who reported exposure to death (within their immediate family) were likely to perceive a lower burden (OR 0.62, 95% CI 0.43-0.89) compared with those who had not. People who reported exposure to a suicide attempt (within their immediate family) were more likely to report favorable attitudes toward the VHS (OR 1.53, 95% CI 1.04-2.25). People who reported exposure to a suicide attempt (by close friends) were more likely to report higher opportunity costs (the extent to which people gave up any benefits, profits, or values when using the VHS; OR 1.77, 95% CI 1.03-3.03) compared with those who did not. People who reported NSSH (within their immediate family) were more likely to report higher levels of ethicality (fit with personal values; OR 1.53, 95% CI 1.02-2.30), self-efficacy (OR 1.51, 95% CI 1.00-2.27), and intervention coherence (confidence in understanding the intervention and how it works; OR 2.10, 95% CI 1.35-3.27), compared with those who did not. People who reported NSSH (by close friends) were more likely to report higher levels of intervention coherence (OR 1.53, 95% CI 1.03-2.27), compared with those who did not.

### Qualitative Analyses of Open-Ended Comments

#### Overview

In all, 340 participants provided at least one open-text comment in response to the TFA items. After responses that were deemed invalid (eg, participants responding with *no comments*) were removed, all of the remaining responses (n=360 comments) were analyzed. Explanatory themes with illustrative quotes were presented within each TFA domain. With respect to opportunity costs, 38 comments were provided. The majority of comments were related to a lack of clarity with respect to this question. Consequently, the participants were unable to provide responses for this domain.

#### Affective Attitude

In total, 9.2% (33/360) of the comments were provided with respect to how good or bad participants felt about taking part in the intervention. Comments within this domain focused on *refinements to the intervention* and *the perceived relevance of situations and responses.* First, the suggested refinements were mainly with respect to general comments about the wording of the situations and responses. In particular, some participants suggested that some of the statements might induce negative thoughts about self-harm and act as a reminder of personal experiences of self-harm:

It brought back a lot of negative memories and feeling surrounding self-harm. Someone would need to be in a decent place in order to use it.

P552, female

Second, participants made reference to the importance of the intervention containing relevant situations to their own experiences where the urge to self-harm may be heightened and responses that are more appropriate. Some participants expressed a desire for more relevant options, as some participants had difficulty relating to either the situations or responses, and they did not link directly to their experiences:

I felt slightly frustrated. I don’t know why. Maybe because I wanted more options.

P348, male

I felt that many of those situations were too specific and did not fit my experiences.

P57, female

#### Burden

In total, 21.6% (78/360) of the comments were provided with respect to the perceived effort required to use the VHS. Comments within this domain focused on *perceptions of usability* and *perceived technology-based challenges*. First, some participants reported specific features of the VHS that made it easy to use, including encouraging and promoting self-reflection as an important feature of the intervention. However, 13 participants reported the psychological effort required to engage with the VHS, such as difficulties in choosing an appropriate situation and response. Participants also described how situations or responses that were perceived to be not personally relevant added to the effort required to engage with the intervention: “Too much effort may result in people giving up completing it.”

Second, participants described potential challenges of using a web-based platform that should be considered in future iterations of the intervention. First, due to the intervention being delivered on the web, 11 participants expressed technical or formatting difficulties that made it difficult to engage with the VHS. Technical issues included difficulties viewing on a mobile device or difficulties in selecting situations and responses from the drop-down menus. These were deemed minor and could be resolved in future iterations of the intervention:

I think it was just this interface made it difficult to scroll. I’m sure if it was on a different site it would be fine.

P544, female

I am doing it on the phone so is difficult to read sometimes.

P339, male

#### Ethicality

In total, 19.2% (69/360) of the comments were provided with respect to the extent to which using the VHS a good fit with an individual’s personal values. Comments within this domain focused on *the perceived strengths of the intervention* and the *perceived importance of having modifiable statements*. First, participants reported that they recognized the situations and responses and indicated a good fit with their personal values. One participant reported that the practical approach to trying to reduce self-harm was a particular strength of the intervention. Another participant described how the intervention helped them focus on more positive thoughts when forming implementation intentions:

It identified to me other ways that I could keep thoughts positive.

P571, female

Practical without being patronising or guilt inducing.

P348, male

Second, there were a small number of participants, who responded negatively to the VHS in its current form; 15 participants perceived the situations and responses as a poor fit with their personal values (such as some of the solutions focusing on other people). In total, 6 respondents suggested acknowledging other situations and responses would strengthen the intervention, as would the feature to add new situations and responses or amend existing ones:

It makes the assumption that self harming is always bad.

P437, female

Like I said it makes me think I should be guilty for wanting to self harm.

P101, female

I don’t really feel comfortable using other people as a solution I think examples of what to do instead (eg go for a walk) could be a way in the moment to realise thinks that might help.

P709, female

Self harming for attention/to hurt or worry others is attention seeking. There are other ways to seek attention. My own self harm was entirely hidden.

P417, male

I’d prefer something a bit more free-wheeling where I could add my own responses.

P254, female

#### Intervention Coherence

In total, 11.1% (40/360) of the comments were provided with respect to how confident respondents were that they understood the VHS and how it worked. Comments within this domain focused on *perceived clarity about the purpose of the intervention* and *perceived confidence in engaging with the intervention correctly.* First, 9 participants reported that they understood the intervention and were positive about its usefulness and helpfulness. However, the majority of the comments related to the need for improved clarity about the purpose of the VHS and how it works:

I believe I’m emotionally fairly intelligent and quite reflective so I believe I understood it fairly well.

It was easy to understand but I imagine that some people would think they could only choose one option.

P427, male

Second, for some participants, a lack of clarity in how the VHS was described affected their confidence in using the intervention in the correct way. A need for clearer instructions for the VHS and, in particular, to help describe how the VHS is intended to work as a way of developing coping plans, was expressed:

The situation and the options were there but no idea how it is supposed to work to stop something that is not tailored towards a specific problem.

P22, male

I wasn’t sure if I should have read all the options even the situations were not applicable to me. I apologise if I got that wrong.

P387, male

#### Perceived Effectiveness

In total, 18.1% (65/360) of the comments were provided with respect to how confident respondents were that the VHS is likely to achieve its purpose. Comments within this domain focused on *perceptions of when the intervention could be used* and *perceived receptiveness of people*. First, participants described how the VHS would be particularly helpful for people in specific contexts or under particular circumstances. Some participants believed that it may be helpful for people when the urge to self-harm was not at its height or as a preventative measure for self-harm: “I think in milder moments and before crisis point, it could help refocus the mind to a more positive and less destructive way of thinking.”

Second, participants believed that the effectiveness of the VHS might depend on how receptive people are to the intervention, and they believed it would be effective for their own circumstances. Some participants expressed concerns that the purpose of the VHS was unclear and expressed a need for further guidance. Participants described how the intervention may induce negative emotions for some people, which may affect how people engage with the intervention:

I think for someone who self harms on instinct it would be useful to have solutions written down as a reminder.

P249, female

It requires a level of concentration which may be difficult to achieve if severely depressed.

P422, male

#### Self-efficacy

In total, 10.3% (37/360) of the comments were provided with respect to how confident respondents were about using the VHS. Comments within this domain focused on *perceptions of personal relevance* and *factors associated with engagement*. First, participants reported that the intervention was easy to use and navigate. However, others have reported that the likelihood of people using or needing a VHS may be dependent upon the relevance of the situations and solutions:

I felt like I learnt to use it. In some cases the situations and options did not fit “me.”

P549, male

Perceptions of personal relevance were perceived to affect confidence in using the intervention. In some cases, participants reported that they had lower confidence in using the VHS because of some of the options not being relevant to their own circumstances; 3 participants expressed doubts that they were using the VHS correctly. Furthermore, one participant also described how motivation to engage with the intervention may differ depending on the situation presented:

Not sure on some of the questions, so put nearest guess to what I might do, most likely I would have selected a different option not available.

P22, male

Wasn’*t* 100% sure about whether or not I could add the same motivation to different situations (I didn’t).

P254, female

## Discussion

### Principal Findings

This paper describes the acceptability of a brief intervention based on implementation intentions to prevent and reduce self-harm. This is the first study to (1) apply the TFA to examine the acceptability of preventative strategies for self-harm, (2) examine for whom and under what circumstances the VHS is more or less acceptable for preventing and reducing self-harm, and (3) develop a series of recommendations for how the VHS can be used to support people to reduce repeated self-harm. This study has four key findings. First, the VHS was rated favorably on five of the seven TFA domains (*affective attitude*, *self-efficacy*, *opportunity costs*, *intervention coherence*, and *perceived effectiveness*). In particular, there was a high proportion of responses at the upper end of the *self-efficacy* and *intervention coherence* items, which suggests that participants understood the VHS and how it works, and they were confident in their ability to use it correctly. Furthermore, there was a high proportion of responses at the lower end of the opportunity cost item, which suggests that people anticipated no problems forming implementation intentions, nor did they have to give up something else, such as time, to engage with the intervention.

Second, with respect to sociodemographic variables, the TFA domain *burden* appeared to be an important indicator of acceptability of the VHS. In particular, lower levels of perceived burden when using the VHS were more prevalent among younger adults (aged 18-24 years) than among older adults, and among people of White ethnic background, compared with participants from a minority ethnic background. This is consistent with national data showing higher rates of self-harm among younger adults [1,3] and among people of White ethnic backgrounds compared with other ethnic backgrounds [33]. Our intervention may benefit some vulnerable individuals. However, our results suggest that further modifications are required to increase acceptability among older adults and minority ethnic groups with respect to perceived burden. Moreover, higher levels of perceived burden were more prevalent among people who reported having a long-term health condition than among those who did not report having a long-term health condition. This may suggest that having the additional burden of a long-term condition may affect the perceptions of interventions designed to target mental health challenges. Given that 30% of the UK population reported living with a long-term condition and a mental health problem (approximately 4.6 million people) [34], this may consequently be an important target population for future studies.

Third, a history of self-harm was associated with perceived acceptability of the VHS. In our study, people who reported self-harm in the past year were more likely to report higher levels of perceived burden compared with those who did not report self-harm in the past year. The VHS has previously been found to be effective for people recently admitted to the hospital after an episode of self-harm [11]. Consequently, our findings suggest that the VHS could be improved further for people with a longer history of self-harm, to make the VHS less burdensome among people who have self-harmed relatively recently (ie, in the past year).

Fourth, our findings show that exposure to death and suicide is associated with perceived acceptability of the VHS. With respect to immediate family, people who reported exposure to death were more likely to report lower levels of perceived burden compared with those who did not. People who reported exposure to a suicide attempt were more likely to report favorable attitudes toward the VHS. Furthermore, people who reported exposure to NSSH were more likely to report higher levels of ethicality (fit with personal values), self-efficacy (confidence in using the VHS), and intervention coherence (confidence in understanding the intervention and how it works), compared with those who did not. With respect to close family and friends, people who reported exposure to NSSH were more likely to report higher levels of intervention coherence than those who did not. Wider research suggests that exposure to suicidal behavior may increase the risk of suicidal behavior through mechanisms such as imitation or social learning [26]. Our findings suggest that exposure to suicidal behavior may have positive effects on intervention acceptability and receptiveness to interventions, as they alleviate the perceived burden of interventions designed to support people in reducing repeat self-harm.

Overall, both our quantitative and qualitative findings suggest high levels of acceptability of the VHS. Consistent with the assumptions of the TFA that engagement in interventions may be dependent upon acceptability [20], our qualitative findings highlight: (1) factors that may affect engagement with the intervention and (2) suggestions to modify the intervention, which may increase acceptability.

With regard to the factors that may affect engagement with the intervention, there were a number of perceived challenges for participants when engaging with the intervention, which could be addressed in future iterations of the intervention, to further increase acceptability. The most commonly reported problems were related to (1) perceptions that statements might (for some people) induce negative thoughts about self-harm and act as a reminder of personal experiences of self-harm (*attitude*), (2) difficulties choosing an appropriate situation and response due to some being perceived as not personally relevant (*burden*), (3) technical issues including difficulties viewing on a mobile device or difficulties in selecting situations and responses from the drop-down menus (*burden*), and (4) perceptions of how receptive people are to the intervention (*perceived effectiveness*).

With regard to how to modify the intervention, in line with the respective TFA domains, there were a number of suggestions that may increase acceptability. The questions included (1) improved formatting of the intervention (*burden*), (2) the feature to add new situations and responses or amend existing ones (*ethicality*), and (3) clearer instructions and further details about the purpose of the VHS and how it works (*intervention coherence*).

### Implications

Our findings suggest high levels of acceptability of the VHS. Participants reported that the VHS was easy to use, and encouraged and promoted self-reflection as a way of supporting people in avoiding future self-harm (*burden*). Minor technical issues were perceived to increase the intervention burden; however, these were judged by the research team to be minor issues. Interventions for self-care practices delivered web-based have been found to be acceptable and demonstrate some level of efficacy across a number of health conditions, including diabetes [35] and psoriasis [36]. This is important, as it suggests that technology-based interventions are perceived as an acceptable delivery mechanism for interventions targeting better management of long-term health conditions. Interventions for mental health problems delivered web-based are considered to be highly acceptable [37], and with further refinements, our intervention could be further developed and delivered as part of remote health care delivery [38,39]. However, further modifications and co-design are needed to make the VHS less burdensome for older adults and to explore specific requirements for people from minority ethnic groups and people with long-term health conditions. This could include clearer instructions about the purpose of the VHS and how it works, and improved formatting.

Our findings suggest that the VHS may be helpful for people in specific contexts or under particular circumstances. The timing of the last self-harm episode appears to be an important consideration when deploying the tool. The VHS was designed to be used as a support tool when someone has the urge to self-harm or immediately following an episode of self-harm [11]. Our findings suggest that further refinements are needed for the tool to make it less burdensome (ie, requiring a lot of effort to engage with) for people who have self-harmed in the previous year. By making the tool easier to engage with, the tool may also be useful as part of long-term support strategies, such as when the urge to self-harm may not be at their height. It is encouraging that no gender differences in acceptability were found, and thus future research could explore the extent to which brief interventions for self-harm could be delivered by health care professionals, for example, research suggests that more emphasis should be placed on improving self-harm care for patients, with a focus on improving the implementation of self-harm management guidelines [40]. One possible route is to explore the use of the VHS alongside GP care, given that the recognition of primary care is an important place to potentially help people reduce repeat self-harm [41].

### Strengths and Limitations

Previous studies examining the prevalence of self-harm have focused on general population samples [29] or adolescent samples [42]. To our knowledge, this is the first study to characterize a national community sample of adults who have previously self-harmed with respect to demographic variables, history of NSSH, suicidal ideation and suicide attempts, and exposure to death and suicide. This is important because knowing more about this community population allows more targeted preventative strategies for self-harm, with respect to specific subgroups who may benefit the most from interventions.

There was evidence of good acceptability of the VHS in line with the TFA domains. In the absence of a universally agreed criterion for TFA acceptability when deploying quantitative measures, we present an assessment criterion that fully operationalizes each of the seven TFA domains and can be used in future studies to assess perceived intervention acceptability. Further developments may be needed to increase perceived acceptability of the VHS for self-harm among older adults, people from minority ethnic groups, and people with long-term health conditions. This study had some limitations. Participants were identified from a pre-existing sample of the general public who reported a previous history of self-harm and were recruited and incentivized by YouGov to complete the questionnaire. Therefore, the sample may not be fully representative of a community with a history of self-harm. However, YouGov attempted to overcome this by seeking the widest possible variation in terms of demographic characteristics, according to people who reported a history of self-harm.

Due to a lack of available studies among community samples with a history of self-harm, we were unable to determine whether our sample is representative of this population. However, we were able to compare our sample with data from the Adult Psychiatric Morbidity Survey of the general population to compare demographic characteristics and self-harm outcomes among people who reported a history of self-harm. Our sample closely resembled the Adult Psychiatric Morbidity Survey data [29] in terms of gender and age. However, our sample contained a lower proportion of people from a minority ethnic background compared with the national data. Our sample also reported a higher prevalence of suicidal ideation (lifetime and past year), suicide attempts (lifetime), and NSSH (lifetime) compared with national data. We were unable to identify data on self-harm outcomes in the past week and NSSH outcomes in the past week or past year. Finally, due to the lack of an appropriate instrument to measure the seven TFA constructs, the research team developed a measure that could be deployed for the present purposes. It would be valuable to conduct follow-up work that examines the psychometric properties of the tool to determine whether it could be used in the context of other contexts.

### Conclusions

A brief intervention based on implementation intentions has been shown to be effective in reducing self-harm in people recently admitted to the hospital after an episode of self-harm. Our findings show high levels of acceptability more generally in people who have previously self-harmed, particularly among younger adults, people of White ethnic backgrounds, and people without long-term health conditions. The intervention still has room for improvement with respect to further modifications around language and technical features, but with emphasis on other target populations. It is hoped that this intervention will provide a useful tool for both individuals to construct their own personalized implementation intentions and as part of long-term support for preventing self-harm delivered by health care professionals.

## Appendix

Multimedia Appendix 1 Proportion of responses at each point according to Theoretical Framework of Acceptability domains.

## Abbreviations

NIHR: National Institute for Health Research
NSSH: nonsuicidal self-harm
PPI: patient and public involvement
TFA: Theoretical Framework of Acceptability
VHS: volitional help sheet
