# Supplementary material for: Acceptability of a Brief Web-Based Theory-Based Intervention to Prevent and Reduce Self-harm: Mixed Methods Evaluation
Source: J Med Internet Res. 2021 Sep 14;23(9):e28349. doi: 10.2196/28349 (PMC8479604; doi:10.2196/28349)
Supplement: Multimedia Appendix 1 [file jmir_v23i9e28349_app1.docx]

**Multimedia Appendix 1.** Proportion of responses at each point according to Theoretical Framework of Acceptability domains.

Table 3. Proportion of responses at each point according to TFA domains

| Item | Reponses at each point (*n*; %) | | | | | | | | | | | | | | | | | | | | | | Total |
| --- | --- | --- | --- | --- | --- | --- | --- | --- | --- | --- | --- | --- | --- | --- | --- | --- | --- | --- | --- | --- | --- | --- | --- |
|  | 0 | | 1 | | 2 | | 3 | | 4 | | 5 | | 6 | | 7 | | 8 | | 9 | | 10 | |  |
| Attitude | 17 | (3.6) | 8 | (1.7) | 21 | (4.4) | 36 | (7.6) | 29 | (6.1) | 172 | (36.1) | 44 | (9.2) | 56 | (11.8) | 40 | (8.4) | 21 | (4.4) | 32 | (6.7) | 476 |
| Burden | 49 | (10.4) | 21 | (4.5) | 28 | (5.9) | 44 | (9.3) | 25 | (5.3) | 80 | (17.0) | 53 | (11.3) | 68 | (14.4) | 56 | (11.9) | 25 | (5.3) | 22 | (4.7) | 471 |
| Ethicality | 39 | (8.4) | 20 | (4.3) | 45 | (9.7) | 48 | (10.3) | 29 | (6.2) | 113 | (24.3) | 40 | (8.6) | 52 | (11.2) | 39 | (8.4) | 20 | (4.3) | 20 | (4.3) | 465 |
| Self-efficacy | 13 | (2.8) | 18 | (3.8) | 16 | (3.4) | 24 | (5.1) | 32 | (6.8) | 101 | (21.6) | 40 | (8.5) | 63 | (13.5) | 64 | (13.7) | 35 | (7.5) | 62 | (13.2) | 468 |
| Opportunity costs | 149 | (33.9) | 37 | (8.4) | 23 | (5.2) | 42 | (9.5) | 18 | (4.1) | 106 | (24.1) | 19 | (4.3) | 16 | (3.6) | 15 | (3.4) | 9 | (2.0) | 6 | (1.4) | 440 |
| Intervention coherence | 14 | (3.0) | 10 | (2.2) | 16 | (3.4) | 25 | (5.4) | 25 | (5.4) | 84 | (18.1) | 47 | (10.1) | 57 | (12.3) | 77 | (16.6) | 34 | (7.3) | 76 | (16.3) | 465 |
| Perceived effectiveness | 39 | (8.4) | 10 | (2.1) | 29 | (6.2) | 31 | (6.6) | 25 | (5.4) | 116 | (24.8) | 64 | (13.7) | 60 | (12.8) | 55 | (11.8) | 16 | (3.4) | 22 | (4.7) | 467 |
